# A Mitophagy-Related Gene Signature for Subtype Identification and Prognosis Prediction of Hepatocellular Carcinoma

**DOI:** 10.3390/ijms232012123

**Published:** 2022-10-12

**Authors:** Chang Liu, Zhen Wu, Liping Wang, Qian Yang, Ji Huang, Jichang Huang

**Affiliations:** 1 Institute of Geriatric Cardiovascular Disease, Chengdu Medical College, Chengdu 610083, China; 2 State Key Laboratory of Genetic Engineering, Department of Biochemistry and Biophysics, School of Life Sciences, Fudan University, Shanghai 200437, China; 3 Key Lab for Arteriosclerology of Hunan Province, International Joint Laboratory for Arteriosclerotic Disease Research of Hunan Province, Department of Pathophysiology, Institute of Cardiovascular Disease, Hengyang Medical School, University of South China, Hengyang 421009, China

**Keywords:** hepatocellular carcinoma, prognosis, mitophagy, mitophagy-related signature, biomarkers

## Abstract

Globally, hepatocellular carcinoma (HCC) is the sixth most common cancer. In this study, the correlation between mitophagy and HCC prognosis was evaluated using data from The Cancer Genome Atlas (TCGA). Clinical and transcriptomic data of HCC patients were downloaded from TCGA dataset, and mitophagy-related gene (MRG) datasets were obtained from the Molecular Signature Database. Then, a consensus clustering analysis was performed to classify the patients into two clusters. Furthermore, tumor prognosis, clinicopathological features, functional analysis, immune infiltration, immune checkpoint (IC)-related gene expression level, tumor stem cells, ferroptosis status, and N6-methyladenosine analysis were compared between the two clusters. Finally, a mitophagy-related signature was developed. Two clusters (C1 and C2) were identified using the consensus clustering analysis based on the MRG signature. Patients with the C1 subtype exhibited upregulated pathways with better liver function, downregulated cancer-related pathways, lower cancer stem cell scores, lower Tumor Immune Dysfunction and Exclusion scores (TIDE), different ferroptosis status, and better prognosis compared with the patients with the C2 subtype. The C2 subtype was characterized by the increased grade of HCC, as well as the increased number of immune-related pathways and m^6^A-related genes. Higher immune scores were also observed for the C2 subtype. A signature containing four MRGs (*PGAM5, SQSTM1, ATG9A,* and *GABARAPL1*) which can accurately predict the prognosis of HCC patients was then identified. This four-gene signature exhibited a predictive effect in five other cancer types, namely glioma, uveal melanoma, acute myeloid leukemia, adrenocortical carcinoma, and mesothelioma. The mitophagy-associated subtypes of HCC were closely related to the immune microenvironment, immune checkpoint-related gene expression, cancer stem cells, ferroptosis status, m^6^A, prognosis, and HCC progression. The established MRG signature could predict prognosis in patients with HCC.

## 1. Introduction

Liver cancer is a major global health challenge, and according to the World Health Organization, it is estimated to affect more than 1 million individuals annually by 2030 [1,2]. Primary liver cancer mainly includes hepatocellular carcinoma (HCC), intrahepatic cholangiocarcinoma (ICC), and combined HCC–cholangiocarcinoma (cHCC-CC) [3,4,5,6]. Among these types, HCC is a life-threatening malignant tumor originating from hepatocytes and is the most common type of primary liver cancer (90%) and the fourth most lethal tumor worldwide, with its preponderance being twice in men than in women [1,2]. The majority of HCCs occur in patients with underlying liver disease, mostly resulting from hepatitis B or C virus infection, alcohol abuse, or nonalcoholic fatty liver disease (NAFLD) [2,3,4,5,6]. HCCs usually develop from cirrhosis and then progress to early and late HCCs. Both early and progressed HCCs can be classified into two main molecular subtypes: proliferation and nonproliferation. The proliferation subtype is characterized by high tumor grade (poor cell differentiation), high alpha-fetoprotein (AFP) levels, poor clinical outcomes, and dismal prognosis [2,3,4,5,6,7]. In the HCC clinical management, providing accurate risk stratification is critical for defining tumor prognosis and treatment recommendation information, which depends on cancer histopathology and stage. Nevertheless, patients with the same histopathology and tumor stage exhibit different prognoses and clinical outcomes, which cannot be easily explained [8,9]. Moreover, the 5-year survival rate of HCC patients is low because of the high recurrence and metastasis rates. Although multiple staging and prognostic systems have been developed, none are universally applicable or agreed upon for predicting survival, and most of them do not include effective biomarkers [10,11,12]. Thus, determining novel and effective biomarkers associated with the prognosis of HCC patients at an early stage is essential.

Mitophagy is a special type of autophagy occurring in mitochondria that specifically clears damaged and old mitochondria, thereby ensuring the quantity and quality of the mitochondrial population in cells [13,14]. When the mitochondria are defective, some special receptors would recognize them showing “eat me”, followed by the generation of the isolation membrane/phagophore surrounding the defective mitochondria as an autophagosome for selective degradation [15]. Depending on the cell types, many signaling pathways are involved in mitophagy (e.g., PINK1/Parkin, BNIP3/Nix, and FUNDC1 signaling pathways) [13]. Many studies have reported that mitophagy is usually associated with many diseases because of its protective mechanisms [16,17,18,19,20]. However, interestingly, mitophagy has “double-edged sword” effects in cancer cells, either promoting or suppressing tumor progression. On the one hand, mitophagy removes dysfunctional mitochondria and protects cells from intracellular oxidative and genotoxic stress, thereby preventing tumorigenesis, which is typically common in early stage tumors. On the other hand, once a tumor forms, tumor cells undergo mitophagy to reduce oxidative stress and provide cycled substrates for cellular proliferation and survival. This phenomenon is more common in higher grade tumors or malignancies. Mitophagy has been proven to be linked to metabolic reprogramming, anticancer therapy resistance, and cancer cell stemness [21,22,23]. Thus, based on the role played by mitophagy in cancer, identifying mitophagy biomarkers would be beneficial in targeting tumors, and this may be an alternative and precise approach with higher anticancer efficiency and fewer side effects.

In this study, the mitophagy profile and clinical value in HCC patients were investigated using The Cancer Genome Atlas (TCGA) sequencing data. Based on the consensus clustering analysis of gene expression profiling, the patients were classified into two robust clusters having significant differences in molecular features and tumor prognosis. Moreover, a mitophagy-related gene (MRG) signature was developed to evaluate the prognosis of these patients in TCGA dataset. Our analysis showed a significant association between the MRG signature and the prognosis, which could act as an independent clinical pathological prognostic factor. In summary, our study revealed that mitophagy-related biomarkers are strongly correlated with the clinical prognosis of patients with HCC.

## 2. Results

### 2.1. Tumor Classification Based on MRGs

**Figure 1:**
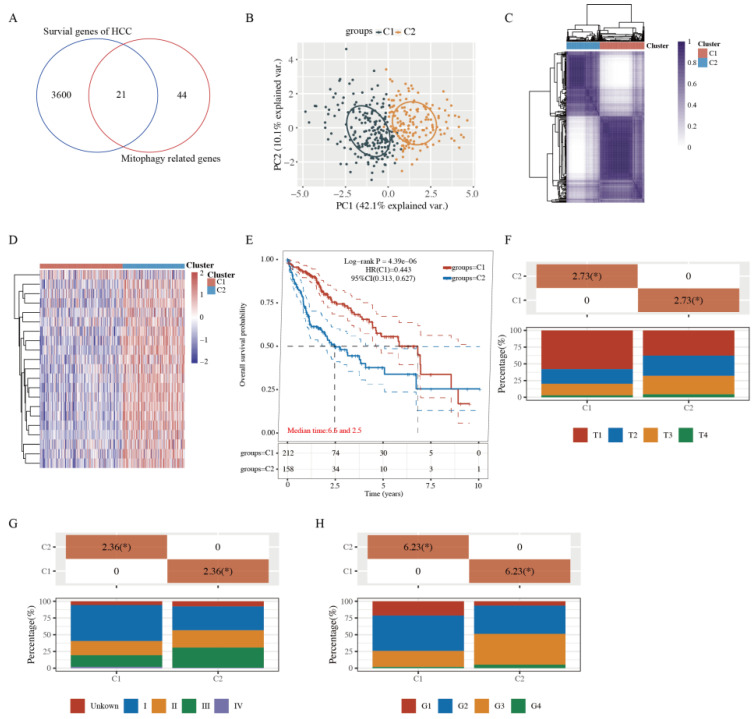
Tumor classification based on the mitophagy-related genes. (**A**) 21 mitophagy- and prognosis-related genes between these two groups. (**B**,**C**) Principal components analysis (PCA) (**B**) and consensus-clustering matrix (**C**) of 371 samples from the TCGA dataset for k = 2. (**D**) Heatmap of mitophagy-related gene expression in different clusters (C1, C2). Red represents high gene expression, and blue represents low expression. (**E**) The Kaplan–Meier curve of overall survival of patients with HCC in two clusters. (**F**) The degree of progression of the primary tumor (T) at diagnosis in the two clusters. (**G**) Distribution of stage at diagnosis in the two clusters. (**H**) The grades (**G**) of tumors in the two clusters. * indicates a significant difference in the distribution of the clinical features between the two groups (*p* < 0.05).

To identify the functional roles of mitophagy status in HCC patients, 3621 HCC prognostic genes were identified through the single factor Cox analysis, and the MRGs were downloaded from the Molecular Signature Database. Next, Venn diagrams were generated to obtain 21 prognosis-related MRGs between these two gene sets (Figure 1A and Supplementary Table S1).

To further explore whether the expression of 21 MRGs was associated with HCC subtypes, these 21 genes were applied to classify TCGA-HCC patients into different subtypes through a consensus clustering analysis. When the clustering variable (kappa) was defined as 2, TCGA-HCC patients could be divided into two clusters based on the results from the principal component analysis (PCA) and a consensus clustering matrix analysis (Figure 1B,C). Furthermore, the MRGs of the two clusters changed significantly (Figure 1D), indicating a better separation effect. The OS time was also compared between the two clusters, and survival curves revealed that cluster 1 (C1) patients had a longer OS time than cluster 2 (C2) patients (HR: 0.443, 95% CI: 0.313–0.627, *p* = 4.39 × 10^−6^, Figure 1E). In addition, C1 patients tended to exhibit no metastases and had relatively early stage tumors (Figure 1F–H).

### 2.2. Functional Enrichment Analysis of Two Clusters

**Figure 2:**
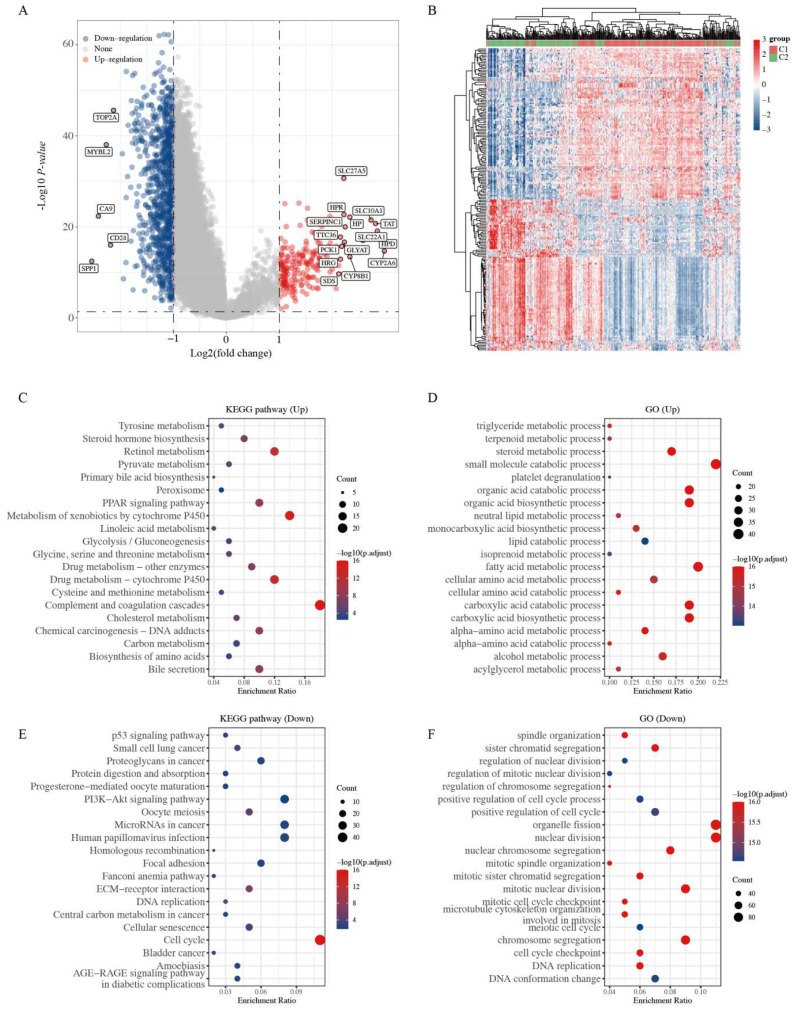
Identification of DEGs between the two clusters grouped by mitophagy-related gene set and functional enrichment analysis. (**A**) Volcano plot of DEGs between two clusters with different mitophagy-related gene expression. The red and blue points represent up- and downregulated genes with statistical significance, respectively. (**B**) Heatmap of the top 50 up- and downregulated genes with the most differential changes. (**C**,**D**) KEGG/GO analysis of DEGs that were upregulated in cluster 1. (**E**,**F**) KEGG/GO analysis of DEGs that were downregulated in cluster 1. DEG: differentially expressed genes.

To further explore the mechanisms underlying the differences between the two clusters, 197 upregulated and 917 downregulated DEGs were identified based on the criteria (C1 vs. C2; fold change ratio ≥2 or ≤0.5 and *p* value < 0.05) (Figure 2A and Supplementary Table S2). The several upregulated genes (*SLC27A5, HPR, SLC10A1, TAT,* etc.) and downregulated genes (*TOP2A, MYBL2, CA9, CD24,* etc.) are labeled in the volcano plot (Figure 2A). The gene expression profiles of the top 50 genes, exhibiting some differences in the two clusters, are presented in a heatmap (Figure 2B).

GO and KEGG enrichment analyses were performed based on these upregulated and downregulated genes (C1 vs. C2). As shown in Figure 2C,E, the pathways of upregulated DEGs were mainly associated with metabolism, primary bile acid biosynthesis, and complement and coagulation cascades, whereas those of downregulated DEGs were mainly related to the p53 signaling pathway, cancer-related pathways, DNA replication, ECM–receptor interaction, and cell cycle. Furthermore, the GO analysis showed similar results, with the upregulated DEGs being mainly correlated with metabolism-related processes, and the downregulated DEGs being mainly related to mitotic nuclear division, positive regulation of the cell cycle, and DNA replication (Figure 2D,F). Studies have demonstrated that activation of the p53 signaling pathway, DNA replication, ECM–receptor interaction, and the cell cycle are cancer hallmarks [24,25,26]. These results suggest that C2 patients display characteristic molecular features of a highly severe stage during HCC development, consistent with the previous clinical stage of HCC patients (Figure 1F–H). This result indicates that C2 patients might exhibit increased tumor cell proliferation and metastasis.

### 2.3. Validation of Genes Associated with the p53 Signaling Pathway and Cell Cycle

**Figure 3:**
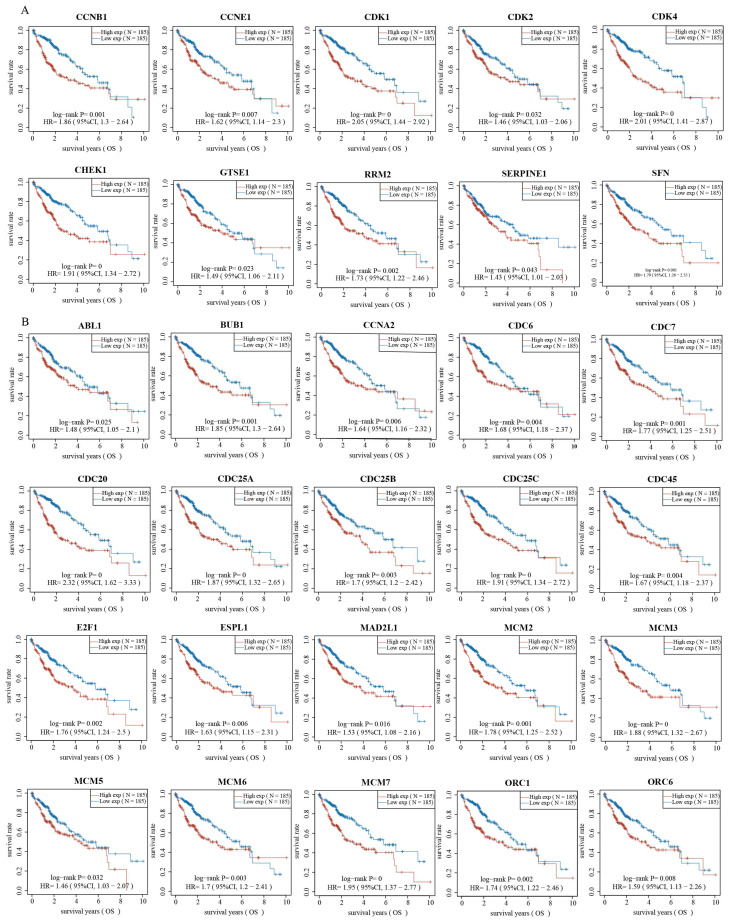
Validation of genes in p53 signaling pathway and cell cycle associated with the prognosis of HCC. (**A**) The Kaplan–Meier curve of overall survival of patients with different expression of genes in p53 signaling pathway. (**B**) The Kaplan–Meier curve of overall survival of patients with different expression of genes in cell cycle.

Based on the functional enrichment analysis results, we further explored whether the gene expression levels of these cancer-related pathways were associated with HCC prognosis. We selected 12 genes related to the p53 signaling pathway and 47 genes related to the cell cycle to validate the correlation. Strikingly, the KM curve revealed that 10 of the 12 genes associated with the p53 signaling pathway, namely *CCNB1, CCNE1, CDK1, CDK2, CDK4, CHEK1, GTSE1, RRM2, SERPINE1*, and *SFN*, led to better OS of HCC patients from the low-expression group compared with those from the high-expression group (Figure 3A). Similarly, the KM curve revealed that the genes associated with the cell cycle led to better OS in patients from the low-expressed group compared with those from the high-expressed group (Figure 3B, Supplementary Figure S1 and Supplementary Table S3). These results suggest that highly expressed genes of the p53 signaling pathway and cell cycle correlate with poor prognosis and outcome, consistent with the pattern of the upregulated genes in high-risk C2 patients (Figure 2).

### 2.4. Comparison of Immune Activity and Tumor Stemness between HCC Subtypes

**Figure 4:**
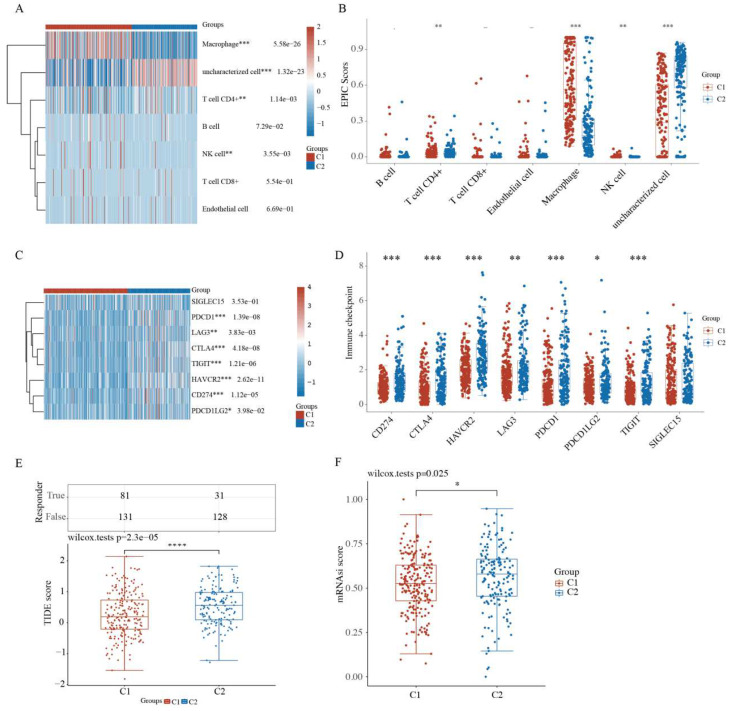
Comparison of the immune activity and tumor stemness between HCC subgroups. (**A**) Immune cell score heatmap by EPIC algorithm. Red represents high expression/score, while blue represents low expression/score. (**B**) Boxplot of immune infiltration status in two clusters by the EPIC algorithm. Heatmap (**C**) and boxplot (**D**) of immune checkpoint-related gene expression between two clusters. Tumor Immune Dysfunction and Exclusion (TIDE) scores (**E**) and cancer stem cell scores (**F**) between two clusters. * *p* < 0.05, ** *p* < 0.01, *** *p* < 0.001, **** *p* < 0.0001.

As a self-limiting system, mitophagy protects cells from the aggravation of inflammation by regulating the adaptive immune response of dendritic cell–T cell synapse, CD8^+^ T cells, and memory NK cells [27,28]. Thus, immune activity and tumor stemness were examined between HCC subgroups. Compared with the C1 subtype patients, the C2 HCC patients exhibited marked differences in the infiltration of immune cells, including macrophages, CD4^+^ T cells, and NK cells (Figure 4A and Supplementary Table S4). The boxplots of immune cells confirmed these differences between C1 and C2 subgroups based on the EPIC algorithm (Figure 4B). Compared with normal tissues, HCC tissues exhibited marked differences in macrophages but not in CD4^+^ T and NK cells (Figure S2A,B).

Checkpoint inhibitors have recently been approved as second-line therapy, which may alter the HCC treatment pattern [29]. The expression of IC-related genes was further analyzed. The heatmap and boxplots revealed that the expression of some genes, including *SIGLEC15, LAG3, PDCD1, CTLA4, TIGIT, HAVCR2, CD274,* and *PDCD1LG2*, was higher in C2 HCC patients than in C1 patients (Figure 4C,D).

Next, based on the expression profiling data, the Tumor Immune Dysfunction and Exclusion (TIDE) algorithm was used as a panel of gene expression signatures to assess distinct immune escape mechanisms in tumors, including the tumor-infiltrating cytotoxic T lymphocyte (CTL) dysfunction and CTL rejection by immunosuppressive factors. A high TIDE score indicated the poor efficacy of the IC blockade (ICB) therapy and short survival following ICB therapy. As shown in Figure 4E, the C2 HCC patients had a higher TIDE score than the C1 patients, consistent with our previous results.

Cancer stem cells (CSCs) are small subpopulations of cancer cells with stem-like features, such as self-renewal, dedifferentiation, high tumorigenic capacity, and distant metastases, leading to cancer recurrence [30,31]. Based on the gene expression profiling of CSCs containing 11,774 CSC-related genes, the C2 HCC patients had higher CSC scores, which suggested the high tumorigenic capacity and tumor cell stemness of the C2 subtype (Figure 4F).

### 2.5. Comparison of Ferroptosis and N6-Methyladenosine between HCC Subtypes

**Figure 5:**
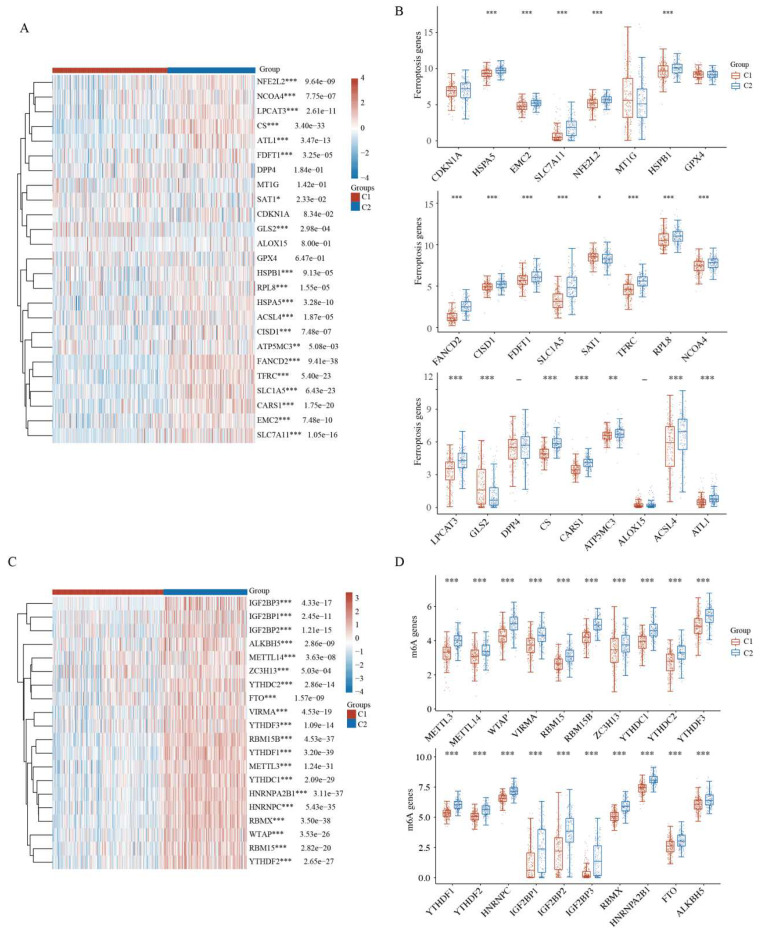
Ferroptosis and N6-methyladenosine analysis between HCC subgroups. (**A**) Heatmap of ferroptosis-related gene expression between two clusters. (**B**) Expression difference of ferroptosis-related genes between the two clusters. (**C**) Heatmap of m^6^A-related gene expression between two clusters. (**D**) Expression difference of m^6^A-related genes between the two clusters. * *p* < 0.05, ** *p* < 0.01, *** *p* < 0.001.

Iron homeostasis is vital for many cellular processes; however, excessive iron accumulation induces reactive oxygen species (ROS) accumulation and oxidative damage. Dysfunction of mitochondrial iron transport has a crucial role in mitochondrial diseases and cancers [30,31]. Mitophagy is related to iron-dependent ferroptosis in cancer. The heatmap and boxplots revealed that 19 of the 25 ferroptosis-related genes, namely *HSPA5, EMC2, SLC7A11, NFE2L2, HSPB1, FANCD2, CISD1, FDFT1, SLC1A5, SAT1, TFRC, RPL8, NCOA4, LPCAT3, GLS2, CS, CARS1, ATP5MC3, ACSL4,* and *ATL1*, were highly expressed in the high-risk C2 subtype compared with the low-risk C1 subtype (Figure 5A,B and Supplementary Table S5). Similarly, 18 of the 25 ferroptosis-related genes were highly expressed in the high-risk HCC group compared with the low-risk healthy group (Figure S3A,B). These results may suggest that mitophagy and ferroptosis are correlated with HCC prognosis.

N6-methyladenosine (m^6^A), the most abundant RNA modification in eukaryotic cells, is closely related to cancer progression, including tumorigenesis, metastasis, and angiogenesis [32,33,34]. The expression of m^6^A-related genes was further analyzed between the two HCC clusters. Strikingly, the heatmap and boxplots revealed that all m^6^A-related genes in the high-risk C2 HCC subtype had higher expression than those in the low-risk C1 HCC subtype (Figure 5C,D). Similarly, 16 of the 19 m^6^A-related genes were highly expressed in the high-risk HCC group compared with the low-risk healthy group (Figure S3C,D). Taken together, HCC prognosis may be correlated with mitophagy and the expression of m^6^A-related genes.

### 2.6. A Prognostic Model Construction in the TCGA-HCC Cohort

**Figure 6:**
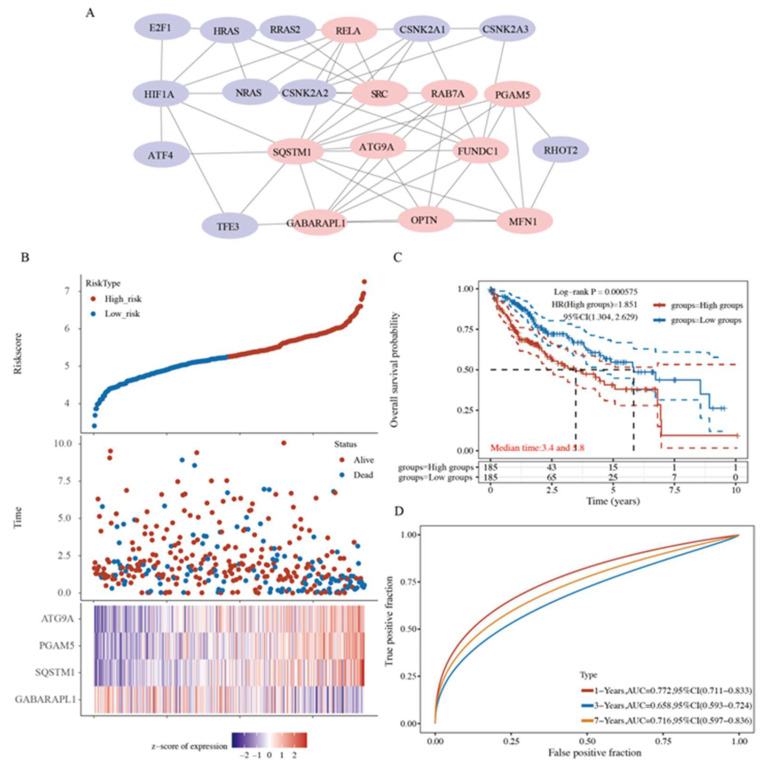
Mitophagy-related prognostic model construction was established for prognosis prediction. (**A**) Top 10 genes were optimized by protein–protein interaction (PPI) networks. (**B**) The risk score of each sample based on the mitophagy-related gene set. Patients were divided into low-risk and high-risk groups according to the median value of the risk score. The high/low expression levels of 4 genes which were involved in the prognostic signature are shown in red/blue in each sample. (**C**) The Kaplan–Meier curve of overall survival differences stratified by signature risk score. (**D**) The receiver operating characteristic (ROC) curves of the signature for overall survival at 1, 3, and 7 years.

Considering the strong association between mitophagy and HCC patient prognosis, we constructed a mitophagy-related prognostic model for prognosis prediction. First, a protein–protein interaction (PPI) network was constructed to optimize the top 10 genes with relatively high confidence, namely *RELA, SRC, RAB7A, PGAM5, SQSTM1, ATG9A, FUNDC1, GABARAPL1, OPTN,* and *MFN1*, by using cytoHubba (Figure 6A and Supplementary Table S6). We performed the step regression analysis to further screen survival-related genes and obtained four MRGs correlated with HCC prognosis, i.e., *PGAM5, SQSTM1*, *ATG9A*, and *GABARAPL1*. The risk score was calculated as follows: risk score = (0.3313 × *PGAM5* expression) + (0.2609 × *SQSTM1* expression) + (0.52 × *ATG9A* expression) + (−0.194 × *GABARAPL1* expression). Based on the median risk score, the HCC patients were equally stratified into the high- and low-risk groups of poor prognosis (Figure 6B). Patients in the high-risk group exhibited a higher mortality rate and lower survival rate than those in the low-risk group (Figure 6B). The KM curves showed that patients in the low-risk group had a better OS than those in the high-risk group (*p* = 0.000575, Figure 6C). A receiver operating characteristic (ROC) analysis was also performed to assess the sensitivity and specificity of our prognostic models. The area under the ROC curves (AUCs) for 1-, 3-, and 7-year OS were 0.772, 0.658, and 0.716, respectively (Figure 6D).

**Figure 7:**
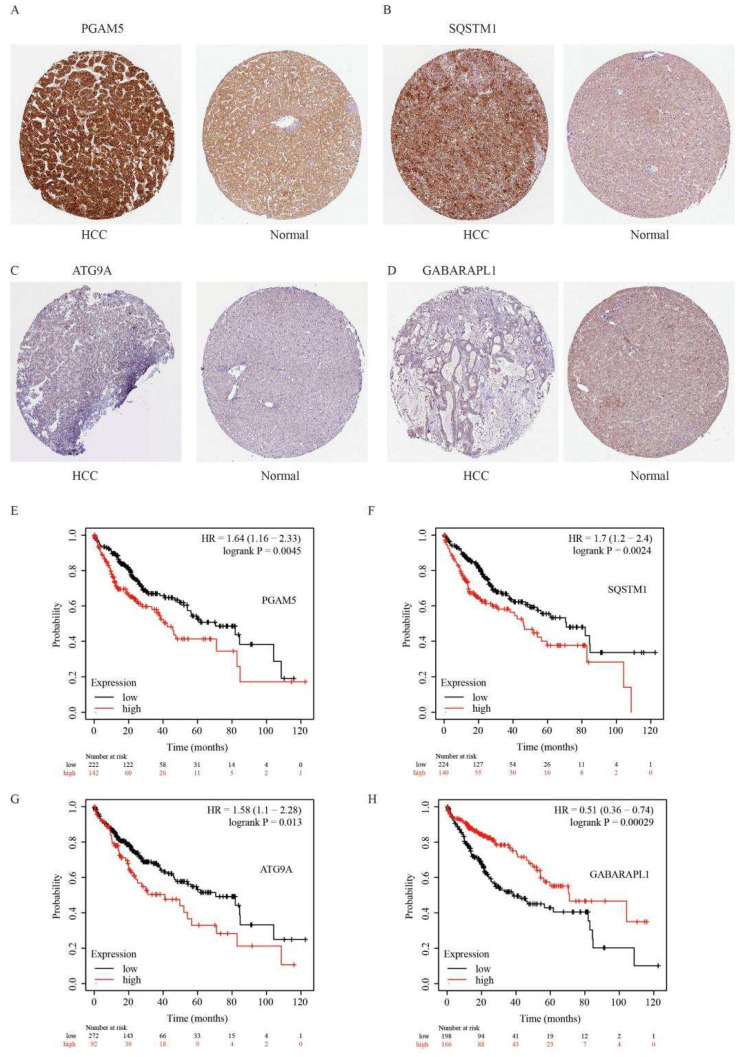
Immunohistochemical results and Kaplan–Meier curve of four genes in HCC tissues and normal tissues. (**A**–**D**) The immunohistochemical expression of four genes in normal tissues and HCC tissues in HPA online database. (**E**–**H**) The Kaplan–Meier curve of overall survival of patients with different expression of four genes in cell cycle.

We further examined the immunohistochemical expression of four genes in normal human tissues and HCC tissues from the HPA database. PGAM5 and SQSTM1 were overexpressed in HCC tissues, and the expression of ATG9A was low in HCC tissues, whereas the expression of GABARAPL1 was absent in HCC tissues but low in normal tissues (Figure 7A–D). In addition, the high expression of PGAM5, SQSTM1, and ATG9A and the low expression of GABARAPL1 predicted poor outcomes in HCC patients (Figure 7E–H).

### 2.7. Four-Gene Signature Validation in Pan-Cancer TCGA Cohorts

To ensure the prediction value of the four genes identified, we performed the step regression analysis for validating the survival-related genes in pan-cancer TCGA cohorts. The four-gene signature was found to exhibit a predictive effect in the other five types of cancer, namely glioma, uveal melanoma, acute myeloid leukemia, adrenocortical carcinoma, and mesothelioma.

**Figure 8:**
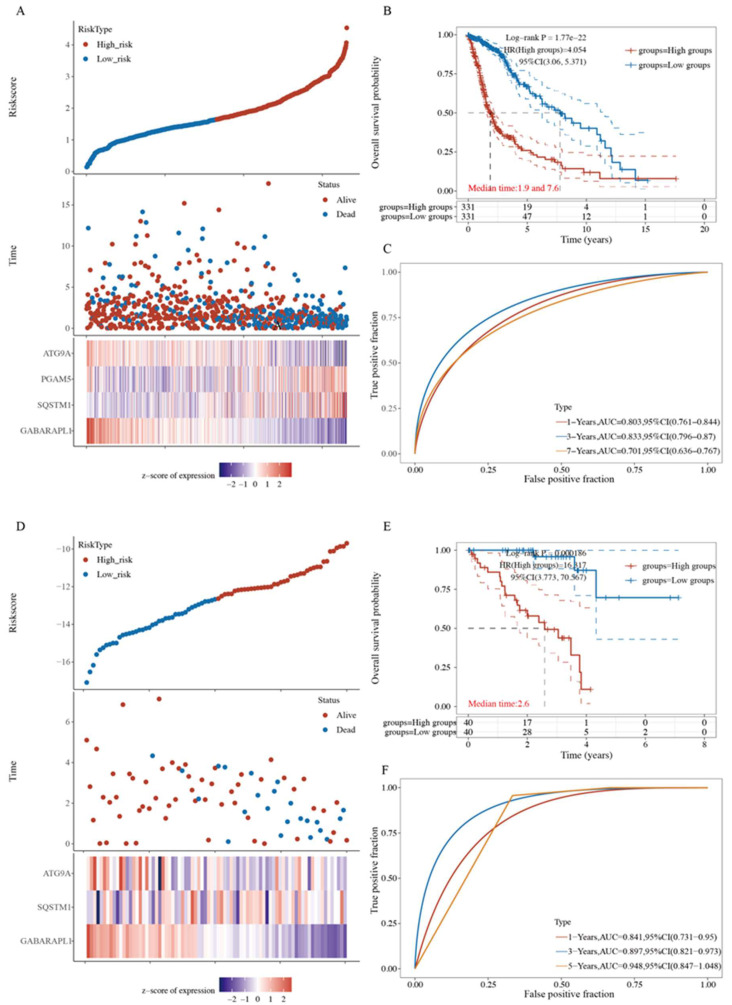
Four-gene signature validations in pan-cancer TCGA Cohort. (**A**) The risk score based on the mitophagy-related gene set in glioma patients. (**B**) The Kaplan–Meier curve of overall survival differences stratified by signature risk score in glioma patients. (**C**) The ROC curves of the signature for overall survival at 1, 3, and 7 years in glioma patients. (**D**) The risk score based on the mitophagy-related gene set in uveal melanoma patients. (**E**) The Kaplan–Meier curve of overall survival differences stratified by signature risk score in uveal melanoma patients. (**F**) The ROC curves of the signature for overall survival at 1, 3, and 5 years in uveal melanoma patients.

For the glioma TCGA cohort, the risk score was calculated as follows: risk score = (0.6457 × *PGAM5* expression) + (0.8062 × *SQSTM1* expression) + (−0.4834 × *ATG9A* expression) + (−0.6285 × *GABARAPL1* expression). The glioma patients were equally stratified into the high- and low-risk groups of poor prognosis (Figure 8A and Supplementary Table S7). The KM curves revealed that patients in the low-risk group had a better OS than those in the high-risk group (*p* = 1.77 × 10^−22^, Figure 8B). AUCs for 1-, 3-, and 7-year OS were 0.803, 0.833, and 0.701, respectively (Figure 8C).

The risk score for the uveal melanoma TCGA cohort was calculated as follows: risk score = (0.8578 × *SQSTM1* expression) + (−1.2004 × *ATG9A* expression) + (−2.0815 × *GABARAPL1* expression). The uveal melanoma patients were equally stratified into the high- and low-risk groups of poor prognosis (Figure 8D). The KM curves showed that patients in the low-risk group had a better OS than those in the high-risk group (*p* = 0.000186, Figure 8E). AUCs for 1-, 3-, and 5-year OS were 0.841, 0.897, and 0.948, respectively (Figure 8F).

The risk score for the acute myeloid leukemia TCGA cohort was calculated as follows: risk score = (0.4655 × *SQSTM1* expression) + (−0.2004 × *GABARAPL1* expression). The acute myeloid leukemia patients were equally stratified into the high- and low-risk groups of poor prognosis (Supplementary Figure S4A). The KM curves revealed that patients in the low-risk group had a better OS than those in the high-risk group (*p* = 0.00868, Figure S4B). AUCs for 1-, 3-, and 5-year OS were 0.567, 0.693, and 0.749, respectively (Supplementary Figure S4C).

The risk score for the adrenocortical carcinoma TCGA cohort was calculated as follows: risk score = (−0.622 × *SQSTM1* expression) + (0.8342 × *ATG9A* expression). The adrenocortical carcinoma patients were equally stratified into the high- or low-risk group of poor prognosis (Supplementary Figure S4D). The KM curves revealed that patients in the low-risk group had a better OS than those in the high-risk group (*p* = 0.00144) (Supplementary Figure S4E). AUCs for 1-, 3-, and 5-year OS were 0.811, 0.849, and 0.801, respectively (Supplementary Figure S4F).

The risk score for the mesothelioma TCGA cohort was calculated as follows: risk score = (0.782 × *PGAM5* expression) + (1.545 × *ATG9A* expression). The mesothelioma patients were equally stratified into the high- and low-risk groups of poor prognosis (Supplementary Figure S4G). The KM curves displayed that patients in the low-risk group had a better OS than those in the high-risk group (*p* = 0.000291) (Supplementary Figure S4H). AUCs for 1-, 3-, and 5-year OS were 0.663, 0.772, and 0.693, respectively (Supplementary Figure S4I).

## 3. Discussion

Hepatocytes are rich in mitochondria, accounting for 13–20% of the liver volume [35]. Mitochondria are double-membrane organelles that have been recognized as the main energy source of ROS production in cells [31]. When mitochondria become dysfunctional, excessive ROS and mitochondrial DNA (mtDNA) are released into the cytoplasm to disrupt cell homeostasis, thus inducing liver cancer development and progression [36]. Mitophagy can reduce intracellular oxidative stress by removing dysfunctional mitochondria, thereby preventing hepatic tumorigenesis, especially in the early stage of cancer. Paradoxically, rapidly expanded liver cancer cells turn over mitophagy for themselves and maintain tumor cell metabolism and nutrient supply for tumor growth and survival, particularly during malignant tumor progression [31,37]. Several studies have reported that agents targeting mitophagy in multiple signaling pathways can prevent hepatocarcinogenesis [38,39,40]. In this study, the association between mitophagy and HCC patient prognosis was evaluated based on RNA-sequencing data from TCGA. Clinicopathological features were significantly correlated with mitophagy, suggesting that mitophagy was closely related to HCC.

Metabolic reprogramming in tumors is a cancer hallmark. Tumor cells usually prefer aerobic glycolysis (Warburg effect) over oxidative phosphorylation (OXPHOS) for cell metabolism. A crosstalk occurs between mitophagy and metabolic rewiring in cancer cells [30,41,42]. Increasing evidence has demonstrated the crucial role of mitophagy in sustaining glycolysis in some cancer types [43,44]. Moreover, tumor cell metabolic reprogramming can activate mitophagy. Our functional enrichment analysis revealed that mitophagy and metabolic status are strongly correlated. The KEGG analysis revealed the upregulation of pyruvate metabolism with related genes enriched in the pathways, except lactate production, indicating that C1 tumor cells may reduce aerobic glycolysis. Moreover, PI3K/AKT signaling drives metabolic reprogramming in cancer cells, including glycolysis [26], which was consistent with our results. Similarly, bile secretion, primary bile acid biosynthesis, and complement and coagulation cascade pathways were also upregulated in C1, implying that the C1 group had a better liver function. A recent study on bladder urothelial carcinoma illustrated that energy metabolism-related pathways and the biological process were upregulated in the group with longer survival, most of which was consistent with our results [45].

The gene *p53* is a TP53-encoded tumor suppressor, which is mutated or deleted in nearly 50% of all human cancers. The tumor suppressor functions of p53 are attributable to the execution of DNA repair, cell cycle arrest, senescence, and apoptosis [24]. The mutated p53 loses the tumor suppression function, and the mut-p53 proteins exhibit a carcinogenic activity independent of wild-type p53 to promote cancer progression, which is termed as the gain-of-function [46]. Moreover, mutation or loss of p53 leads to an increase in the Warburg effect and intracellular oxidative stress, thereby promoting migration and metastasis of cancer cells [24,46,47]. Importantly, a complex molecular circuit exists between p53 and mitophagy involved in cancer cell survival and death. Liu et al. reported that mitophagy restricted the p53 levels in HCC stem cells through the PINK1-mediated signaling pathways to activate the stem cell factor Nanog, indicating that mitophagy could regulate p53 activity [48]. Moreover, whether the cell cycle control is associated with mitophagy has been investigated. Veeriah et al. reported that the expression of the mutated MRG *PARK2* has a role in cyclin E degradation [25,26]. KEGG analysis revealed that the p53 signaling pathway, cell cycle, and cellular senescence pathways were downregulated in the C1 subtype, and GO analysis revealed that the most enriched downregulated biological process was cell division, suggesting a reduction in tumor cell proliferation and metastasis in C1 patients. Furthermore, *p53*- and cell cycle-related genes were extracted to verify the survival curve, which also exhibited a similar patten as the enrichment analysis.

The damage-associated molecular patterns (e.g., mtDNA, ROS, and ATP) from mitochondria could activate immune responses of innate immune cells [37]. Thus, being a self-limiting system, mitophagy guard cells from the aggravation of inflammation by regulating the adaptive immune responses of dendritic cell–T cell synapse, CD8^+^ T cells, and memory NK cells [27,28]. Functional immune infiltration analysis revealed that mitophagy is strongly correlated with immune and inflammatory responses, suggesting the interaction between mitophagy and the tumor immune microenvironment. Mitophagy was significantly correlated with most ICI biomarkers, acting as biomarkers and IC inhibitors, or participated in HCC tumorigenesis and progression. IC inhibitors have recently been approved as second-line therapy, possibly altering the HCC treatment pattern [29]. These results suggest that mitophagy affects the tumor microenvironment through immune cell infiltration, thus mediating HCC carcinogenesis, and plays a vital role in the sensitivity and resistance of immune therapy.

CSCs are small subpopulations of cancer cells, characterized by stem-like features, such as self-renewal, dedifferentiation, high tumorigenic capacity, and distant metastases, resulting in cancer recurrence [30,31]. Mitophagy regulates the CSC subpopulation, as it is involved in the promotion of stemness by causing glycolysis and limiting oxidative metabolism in the extreme environment, as well as induces therapy resistance [31]. Chen et al. reported that BNIP3L-dependent mitophagy induced by the hepatitis B virus × (HBx) protein enhanced the stemness of liver CSCs by regulating glycolytic metabolism [49]. Our results indicated that HCC mitophagy was significantly correlated with CSCs, with the CSC score being higher in the C2 subtype, which implied that tumor cells in C2 had more stemness. A study also proved that mitophagy maintained stemness in hepatic CSCs by restricting p53, which also further helped explain our results [48].

Many cellular processes require iron homeostasis; however, excessive iron accumulation induces ROS accumulation and oxidative damage. Dysfunction of mitochondrial iron transport is crucial in the development of mitochondrial diseases and cancers [30,31]. Mitophagy’s function in iron-dependent ferroptosis is paradoxical in cancer. PINK1/PARK2-dependent mitophagy could suppress pancreatic tumorigenesis by controlling mitochondrial iron metabolism [31]. On the contrary, in colorectal cancer, mitophagy elevates iron accumulation in intestinal epithelial cells and promotes tumor cell death [50]. Among 25 ferroptosis-related genes, the expression of 20 genes was significantly higher, 4 genes showed no difference, and 1 gene was downregulated in the C2 subtype, showing that some of them were correlated with HCC prognosis. The potential mechanism of action of ferroptosis and mitophagy in HCC patients should be further investigated.

m^6^A is the most abundant RNA modification in eukaryotic cells. This modification is closely related to cancer progression, including tumorigenesis, metastasis, and angiogenesis [32,33]. Increasing evidence suggests that m^6^A plays a dual role in cancer depending on cancer types [32,33,34]. On the one hand, m^6^A regulates the expression of oncogenes or tumor suppressor genes, thereby influencing cancer progression. On the other hand, m^6^A levels and m^6^A enzyme expression and activity can be modulated, thereby affecting m^6^A’s role in cancer [34]. HCC progression is related to aberrant m^6^A modification [32,33]. Some m^6^A-related biomarkers such as *METTL3* and *YTHDF2* are upregulated in HCC patients and promote HCC progression [32]. However, some biomarkers such as *METTL14* have an adverse effect on HCC development [33]. A correlation of mitophagy and m^6^A-related genes with HCC prognosis was observed. This study was the first to report an association between mitophagy and m^6^A modulation, warranting additional studies in the future.

Because of the strong association between mitophagy and clinicopathological features in HCC patients, the patients were stratified into high- and low-risk groups of poor prognosis with an established signature. In this study, combined with the use of a step regression analysis, the signature of four genes strongly affected survival prediction. Of the 21 MRGs, *ATG9A*, *PGAM5*, *SQSTM1*, and *GABARAPL1* were differentially expressed at the transcriptomic level between tumor and normal tissues. ATG9 is the only multi-transmembrane protein of the core autophagy machinery delivered to the formed autophagosome by vesicular transport from the trans-Golgi network and/or endosomes, and ATG9A-mediated lipid scrambling is involved in autophagosome expansion [51,52,53].

Phosphoglycerate mutase 5 (PGAM5) is a mitochondrial serine [Ser)/threonine [Thr) phosphatase usually located in the inner mitochondrial membrane and regulates mitochondrial dynamics and programmed cell death [54]. PGAM5 also regulates mitophagy through different signaling pathways, contributing to cellular senescence, neurological diseases, and cancer [55,56,57]. SQSTM1, particularly (SQSTM1)/p62, is a soluble autophagy receptor with LIR motifs and one ubiquitin-binding domain at the C-terminus, which initiates selective autophagy in cells [37,58]. SQSTM1/p62 is overexpressed in renal cell carcinoma due to chromosome 5q amplification and accumulated under pre-oncogenic conditions of pancreatic and hepatic cells [59,60]. Furthermore, p62-induced mitophagy averts mitochondrial dysfunction and leads to poor prognosis in human acute myeloid leukemia [61]. A receptor-associated protein-like 1 (GABARAPL1) belongs to the mammalian ATG8 family of proteins, corresponding to the autophagosome size. GABARAPL1 participates in LC3 recruitment to aid extension of the autophagosome membrane during autophagy [25,62]. GABARAPL1 is suggested to be a tumor suppressor and its lower levels have been reported to be related to a poor prognosis in liver and breast cancer patients [63,64]. This finding is consistent with the results of our study showing that GABARAPL1 exhibited a protective role in HCC. Furthermore, the four genes were examined in the prognostic risk model of another five cancer types; however, they still exhibited a predictive effect, suggesting that these four genes can act as cancer therapeutic targets.

We summarized the similarities and differences between three studies, which have reported the results similar to those of our study. All these studies included patients with HCC. However, our study had some major differences compared with these three studies. First, we used a different method for selecting genes. Xu et al. used 16 survival MRGs to construct the prognosis model [65]. Chen et al. categorized patients into two groups according to the median immune score and searched the MRGs between the groups to construct the prognosis model, but they did not construct mitophagy-related HCC subtypes [66]. Wang et al. identified HCC subtypes based on 26 selection-free MRGs, while our study identified the low-risk C1 subtype and high-risk C2 subtype based on 21 survival-related MRGs [67]. Second, although the three studies selected MRGs by using their own methods, they did not provide deeper insights into the molecular mechanisms underlying the high-risk and low-risk HCC subtypes. We tried to explore the underlying mechanisms of the low-risk C1 subtype and the high-risk C2 subtype, which was the main difference between our study and the other three studies. Compared with the high-risk C2 subtype, the low-risk C1 subtype had upregulated pathways with better liver function, downregulated cancer-related pathways, lower CSC scores, lower TIDE scores, and lower expression of ferroptosis- and m^6^A-related genes. This deepened our understanding of the molecular mechanisms underlying low- and high-risk HCC populations. Third, as the three studies were based on pure biological information analysis without experimental validation, false-positive results with those numbers of genes may be inevitable. Their results also revealed that the types of genes in one study were quite different from another study, except *PGAM5*. In our study, all four selected genes overlapped with those in the three studies, especially *PGAM5*, unexpectedly appearing in two of those studies. This indicates that our results are reproducible and reliable to some extent. A recent study validated *PGAM5* function, thereby providing sufficient evidence to support the reliability and reproducibility of our results [68]. In summary, our study not only has a novelty but also provides further support for previous research.

The present study also has some limitations. The specific role of MRGs in HCC should be further elucidated in vitro and in vivo. Additionally, the specific mechanism of action of mitophagy genes in HCC progression should be explored in further studies, which may provide new strategies for HCC treatment.

In conclusion, mitophagy was found to be closely related to the immune microenvironment, IC-related genes, CSCs, and prognosis in HCC patients. The MRG signature was developed to predict the survival of these patients. In the era of precision medicine, this signature would be an effective strategy to meet the clinical requirements of HCC management to a certain extent.

## 4. Materials and Methods

### 4.1. Data Collection Information of HCC Patients

Data of 371 HCC patients, including RNA-sequencing expression profiles and the corresponding clinical information, were downloaded from TCGA dataset (https://portal.gdc.com (accessed on 12 March 2022)).

### 4.2. Selection of MRGs and Consensus Clustering Analysis

Based on the *p* value, risk coefficient, and univariate analysis of significant gene expression, 3621 HCC prognostic genes were identified through the single-factor Cox analysis conducted using the forest plot-related R packages. MRGs were downloaded from the Molecular Signature Database (MSigDB, http://www.broad.mit.edu/gsea/msigdb/ (accessed on 20 March 2022)). After the removal of overlapping genes, 65 MRGs were retrieved. The Venn online tool was used to select the genes overlapping between 3621 HCC prognostic genes and MRGs. The consensus clustering analysis was then conducted using the “ConsensusClusterPlus” R package (version 1.54.0), with the maximum cluster size set to 6. This heatmap was generated using the “pheatmap” R package (version 1.0.12), which helped visualize the expressions of relatively highly expressed MRGs. Kaplan–Meier (KM) survival curves were generated using the R package “survival.”

### 4.3. Identification of Differentially Expressed Genes and Enrichment Analysis

Differentially expressed genes (DEGs) between groups, which were identified through consensus clustering, were explored, and volcano plots (fold change ≥2.0 or fold change ≤0.5, and *p* value < 0.05) were established to reveal the abundant differential changes by using the “ggplot2” R package. The heatmap was plotted to visualize the expression profiling of DEGs with the top 50 differential changes. To better comprehend the underlying functions of potential targets, we performed enrichment analyses, such as Gene Ontology (GO) and Kyoto Encyclopedia of Genes and Genomes (KEGG), using the “ClusterProfiler” R package (version 3.18.0). Moreover, the OCLR algorithm was used to determine the relationship between mitophagy and cancer stem cells (CSCs) [69].

### 4.4. Immune Infiltration and Immune Checkpoints Analysis

Based on the clusters formed through consensus clustering, immune infiltration of the clusters was performed with EPIC algorithms using the “immunedeconv” R package and visualized in the form of a heatmap and boxplot. Moreover, IC-related genes (*CD274*, *PDCD1*, *PDCD1LG2*, CTLA4, *LAG3*, *HAVCR2*, *TIGIT*, and *SIGLEC15*) were selected [70], and the expression values of these genes were visualized using “ggplot2” and “pheatmap” R packages. Statistical significance was determined using the Wilcox test, and a *p* value < 0.05 was considered significant.

### 4.5. Aberrancies and Functional Implications of Ferroptosis and N6-methyladenosine [m^6^A)

Ferroptosis-related genes were obtained from a previous study [71]. Aberrancies and functional implications of ferroptosis were systematically analyzed between the two clusters. m^6^A-related genes were derived from Juan Xu’s research [72] on the molecular characterization and clinical significance of m^6^A modulators and systematically analyzed between the two clusters. All the aforementioned analysis methods and the R package were implemented by R foundation for statistical computing (version 4.0.3, 2020).

### 4.6. Gene Signature Identification

Protein–protein interactions of MRGs were detected using STRING, and the top 10 of these genes were selected using the built-in CytoHubba App in Cytoscape software. Through univariate Cox proportional hazards regression analysis, we analyzed the HCC dataset to determine whether MRGs could be used to predict overall survival (OS) in HCC patients. Additionally, the multi-factor Cox regression was used to analyze the data, and the step function was used to perform iterations. The optimal model was selected using the “glmnet” R package. KM survival curves were plotted for OS and were compared using the log-rank test. Hazard ratios (HRs) with 95% confidence intervals (Cis) were calculated through a Cox proportional hazards analysis. The HCC gene signature was explored for its predictive effects in pan-cancers. Furthermore, the immunohistochemical expression of four genes were examined in normal human tissues and HCC tissues from the HPA database (https://www.proteinatlas.org (accessed on 15 May 2022)).

## Data Availability

All data are included in the article and Supplementary Materials.

## Supplementary Materials

The following supporting information can be downloaded at: https://www.mdpi.com/article/10.3390/ijms232012123/s1.
