# Supplementary material for: A Mitophagy-Related Gene Signature for Subtype Identification and Prognosis Prediction of Hepatocellular Carcinoma
Source: Int J Mol Sci. 2022 Oct 12;23(20):12123. doi: 10.3390/ijms232012123 (PMC9603050; doi:10.3390/ijms232012123)
Supplement: Supplementary file 1 [file ijms-23-12123-s001.zip › ijms-1909924-supplementary.pdf]

A mitophagy-related gene signature for subtype identification and prognosis prediction of hepatocellular carcinoma

Chang Liu[a,#], Zhen Wu[b,#], Liping Wang[a], Qian Yang[a], Ji Huang, Jichang Huang[a,*]

[a]Institute of Geriatric Cardiovascular Disease, Chengdu Medical College, Chengdu, Sichuan, China

[b]State Key Laboratory of Genetic Engineering, Department of Biochemistry and Biophysics, School of Life Sciences, Fudan University, Shanghai, China

[c]Department of Pathophysiology, Institute of Cardiovascular Disease, Key Lab for Arteriosclerology of Hunan Province, International Joint Laboratory for Arteriosclerotic Disease Research of Hunan Province, Hengyang Medical School, University of South China, Hengyang, China

[#], These authors contributed equally to this work.

[*]Corresponding authors: Jichang Huang, email: huangjichang1988@126.com.

Supplementary figure and figure legends

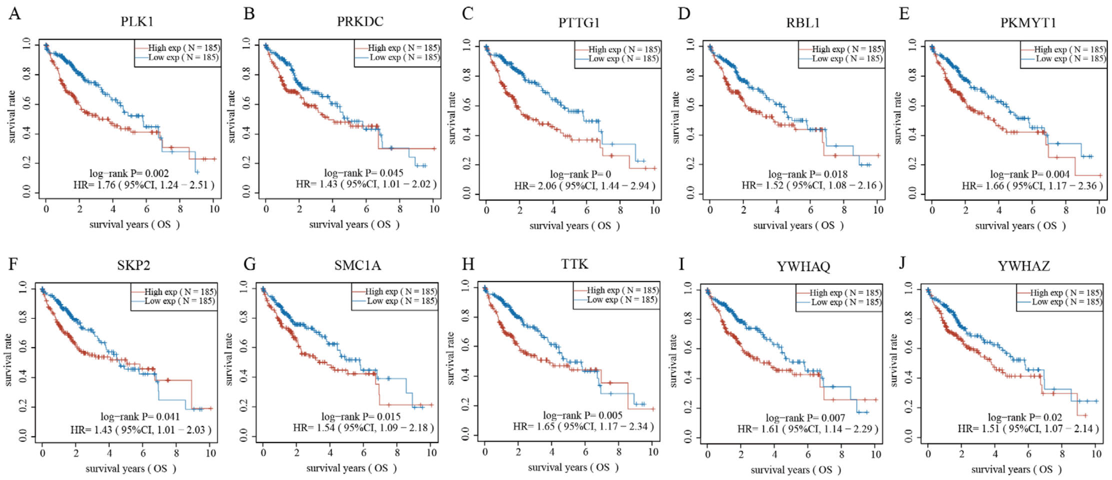

Figure S1 Validation of genes in cell cycle associated with the prognosis of HCC. (A-J) The Kaplan–Meier curve of overall survival of patients with different expression of genes in cell cycle.

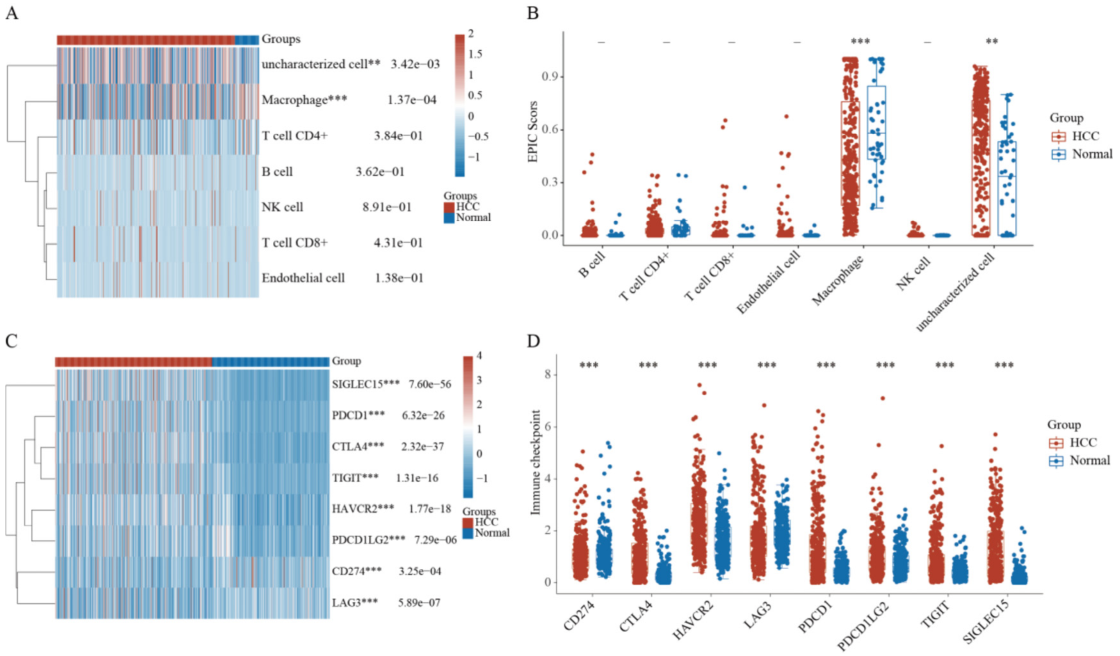

Figure S2 Comparison of the immune activity and tumor stemness between HCC subgroups. (A) Immune cell score heatmap by EPIC algorithm. Red represents high score, while blue represents low score. (B) Boxplot of immune infiltration status in two clusters by the EPIC algorithm. Heatmap (C) and boxplot (D) of immune checkpoint-related genes expression between two clusters.

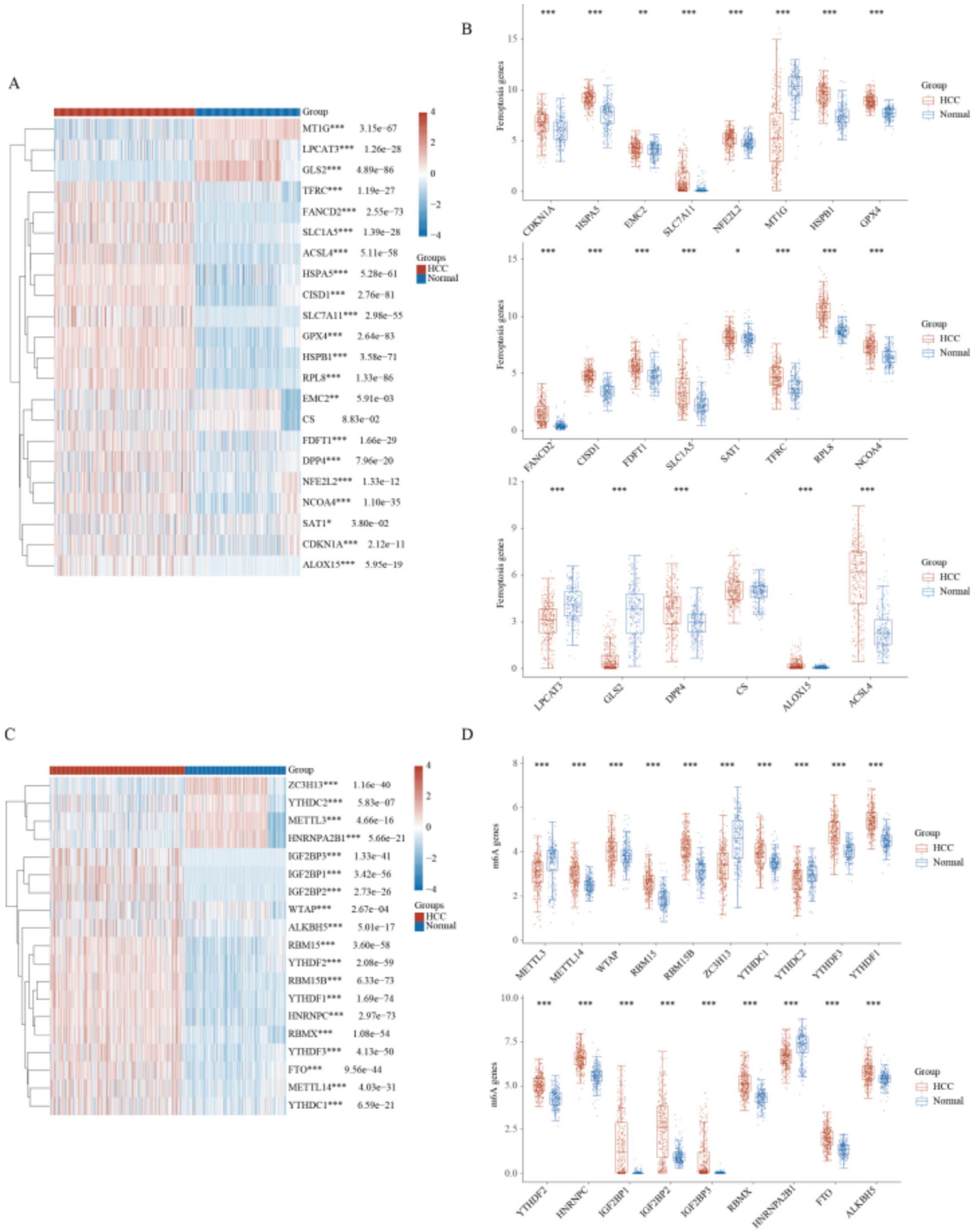

Figure S3 Ferroptosis and N6-me-thyladenosine analysis between HCC and normal tissues. (A) Heatmap of ferroptosis- related gene expression between HCC and normal tissues. (B) Expression difference of ferroptosis- related genes between HCC and normal tissues. (C) Heatmap of m6A-related gene expression between two clusters. (D) Expression difference of m6A-related genes between HCC and normal tissues.

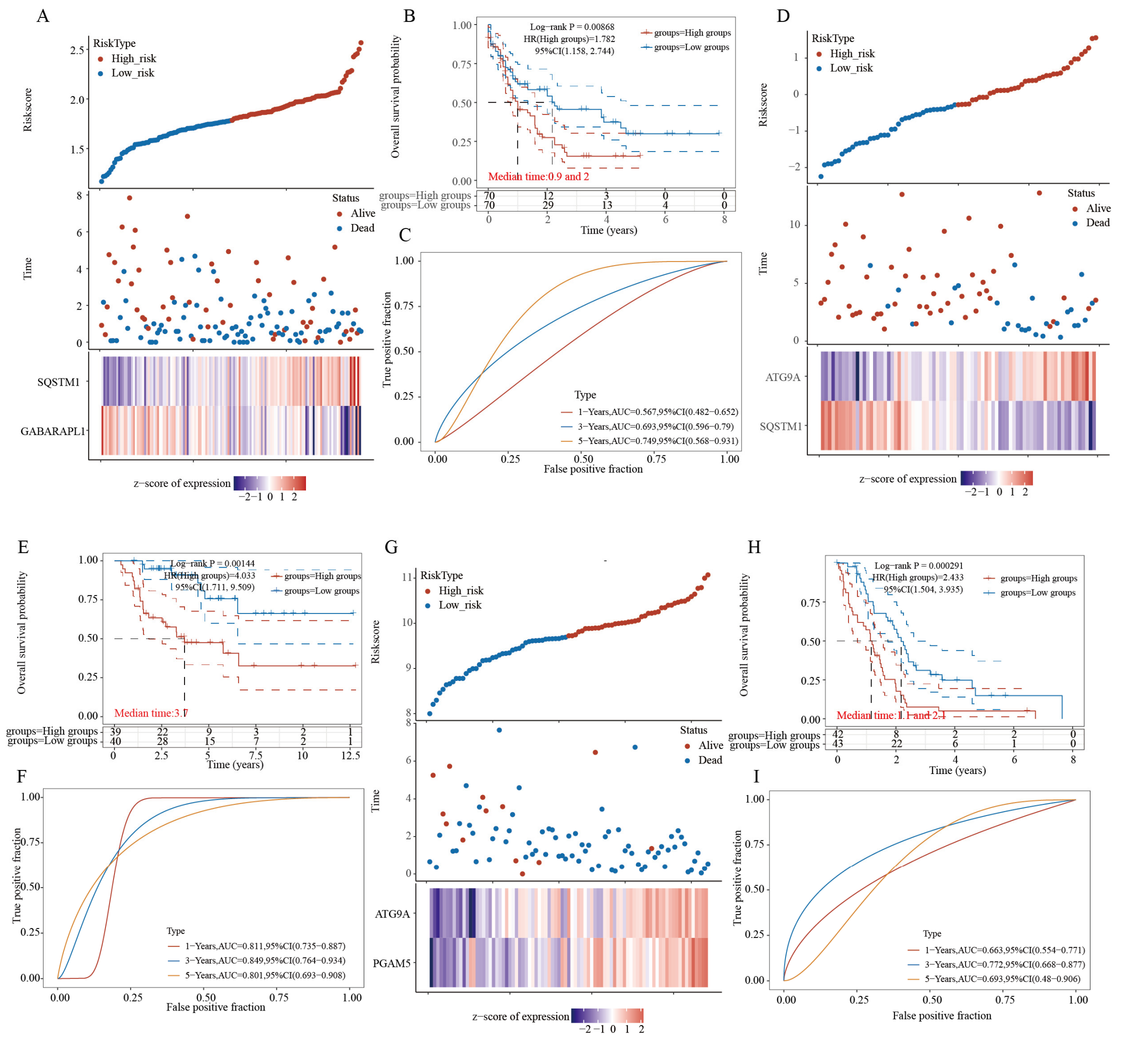

Figure S4 Four-gene signature validations in pan-cancer TCGA Cohort. (A) The risk score based on the mitophagy-related gene set in acute myeloid leukemia patients. (B) The Kaplan–Meier curve of overall survival differences stratified by signature risk score in acute myeloid leukemia patients. (C) The ROC curves of the signature for overall survival at 1, 3, and 5 years in acute myeloid leukemia patients. (D) The risk score based on the mitophagy-related gene set in adrenocortical carcinoma patients. (E) The Kaplan–Meier curve of overall survival differences stratified by signature risk score in adrenocortical carcinoma patients. (F) The ROC curves of the signature for overall survival at 1, 3, and 5 years in adrenocortical carcinoma patients. (G) The risk score based on the mitophagy-related gene set in mesothelioma patients. (H) The Kaplan–Meier curve of overall survival differences stratified by signature risk score in mesothelioma patients. (I) The ROC curves of the signature for overall survival at 1, 3, and 5 years in mesothelioma patients.
